# British Lunatic Asylums
*1. Thirty-second Report of the Friends' Retreat, York, 1849. 2. Report of the Kent County Lunatic Asylum for 1848. 3. Report of the Lunatic Asylum for the North and East Riding of Yorkshire, 1849. 4. Twenty-ninth Report of the Dundee Royal Lunatic Asylum, 1848-9. 5. Report of the Montrose Lunatic Asylum, 1849. 6. Report of the Belfast Lunatic Asylum, 1849. 7. Eleventh Annual Report of the Suffolk Lunatic Asylum, 1849. 8. Twenty-second Annual Report of James Murray's Royal Lunatic Asylum, 1849. 9. Report of the West Riding Lunatic Asylum, 1849. 10. Report of the Crichton Institution, Dumfries, 1849.


**Published:** 1850-01-01

**Authors:** 


### Art. VIII.—BRITISH LUNATIC ASYLUMS.*

The Reports before us contain a faithful record of the progress of our principal British County Asylums, and as such constitute a valuable mass of facts and statistics connected with the present condition of lunacy in this country. From the Report of the superintendent of the Retreat, York, it appears that twenty-three were admitted during the preceding year, and the average number of admissions, for six years past, has likewise been twenty-three. Of the number admitted, one-third were re-admissions, which is a proportion considerably higher than is shown by the average experience of the establishment. The number under care, at one time, has been greater than in any former year, having for some weeks been as high as 117. The average number resident during the year has been 113·08, or three more than during the preceding year. At the present time there are 110 patients in the house.

Of those admitted during the year, five were unconnected with the Society of Friends; and there are at present fourteen patients, not connected with the Society, under care.

During the year, twenty-seven patients have either been discharged, or removed by their friends, or have died. Of this number, one half were discharged recovered. In most of these cases, satisfactory reports, as to the continued recovery of the former objects of our care, have been received.

It appears, "the mean mortality of the establishment since its opening has been about 4·7 per cent.; but during the past year it has amounted to 8 per cent. Fluctuations of this kind will necessarily occur in all establishments; and in the experience of the Retreat, a year of excessive mortality has usually been compensated by a succeeding year in which the deaths have been few. Four of the deaths were those of persons sixty-eight years of age and upwards, and of these one had attained the age of eighty-six years. The causes of death and ages of those who died were as follow:—

* 1. Thirty-second Report of the Friends' Retreat, York, 1849. 2. Report of the Kent County Lunatic Asylum for 1848. 3. Report of the Lunatic Asylum for the North and East Riding of Yorkshire, 1849. 4. Twenty-ninth Report of the Dundee Royal Lunatic Asylum, 1848-9. 5. Report of the Montrose Lunatic Asylum, 1849. 6. Report of the Belfast Lunatic Asylum, 1849. 7. Eleventh Annual Report of the Suffolk Lunatic Asylum, 1849. 8. Twenty-second Annual Report of James Murray's Royal Lunatic Asylum, 1849. 9. Report of the West Riding Lunatic Asylum, 1849. 10. Report of the Crichton Institution, Dumfries, 1849.

| | Male. | | Female. |
|---|---|---|---|
| Diarrhœa | — | ... | 86 |
| Mortification, with slight general Paralysis | 35 | ... | — |
| Cancer | — | ... | 71 |
| Atrophy and gradual exhaustion | — | ... | 68 |
| Exhaustion, supervening rapidly on admission | 56 | ... | — |
| Inflammation of the Lungs, connected with Influenza | 57 | ... | 74 |
| Pulmonary Consumption | 38 | ... | — |
| Softening of the Brain | 36 | ... | — |

"Two of the deaths, it will have been seen, were from pulmonary inflammation, consequent on the influenza, which prevailed epidemically at the end of last year. The female who died was seventy-four years of age, and had frequently suffered from pleuritic inflammation. In the other case, there was great debility, from slight general paralysis and diarrhœa. During the prevalence of influenza, more than half the patients suffered from more or less severe attacks of the epidemic. For the most part, however, the disease assumed its less severe form. One remarkable feature of this epidemic, as observed at the Retreat, was that of its prevailing almost exclusively amongst the female inmates—patients and nurses—not more than three or four of the male inmates coming under treatment at all."

In the report of the Kent County Asylum, which, we are glad to hear, is in so flourishing and excellent a condition, it is observed, when speaking on the subject of restraint,—

"It has been deemed necessary to apply restraint to the person in two instances only during the year; of a female for twenty-one hours, and of a male for forty-eight hours. In the case of the latter, the strait waistcoat (which was the kind used) was removed at the end of every twelve hours, and a short time allowed to afford freedom to the arms and a change of position.

'The female restrained is the same who was also restrained the year before (mentioned in the Report), and to prevent the like disaster of self-inflicted injury to her person. She had badly lacerated her breasts, and made attempts to tear out her tongue, by which that organ was injured.

"The male who was likewise restrained is an epileptic, and was on this occasion seized with a paroxysm of maniacal excitement, in which, at the onset, he terribly bruised and wounded himself; whilst his attendant had a narrow escape from injury with a weapon which the patient had obtained by the destruction of the interior of his room.

"But the only benefit derived from restraining, in this case, was not merely the prevention of mischief and personal injury, since, under its use, we were enabled to apply remedies which it is believed were serviceable in shortening the attack."

In the *Twenty-ninth Report of the Lunatic Asylum for the North and East Ridings of Yorkshire* (1849), we find the following statistical statement:—

"There were in the Asylum on the 30th of March last,

| | | |
|---|---|---|
| | 74 males, | 57 females. |
| Admitted since . . | 30 „ | 22 „ |
| | 104 | 79—together, 183 |

who have been under treatment, of whom there have been finally

| | | |
|---|---|---|
| Discharged . . . . . . | 12 males, | 8 females. |
| Out on trial . . . . . . | 4 „ | 1 „ |
| Improved . . . . . . | 2 „ | 0 „ |
| Died . . . . . . . . | 9 „ | 1 „ |
| Remaining under treatment | 77 | 69—total, 146. |

The subjoined facts are interesting, as illustrative of the benefit of moral treatment in certain cases of insanity:—

"A young man who had been apprenticed to a wheelwright, and whose father is insane, was admitted in a state of violent mania, which left him in a condition of the lowest mental capacity. When roused from his apathy, it was to commit some improper act, or to attempt to escape. He was entirely heedless of his personal comforts, and his habits would soon have degenerated into the worst description. Many trials were made to stimulate him to useful exertion in the garden. No sooner was he engaged in the carpenter's shop, than his intellects began to brighten; he made a wheelbarrow for the bricklayers, and commenced a pair of wheels for a water-cart: his recovery was very rapid, he was discharged cured, and has remained well upwards of a year. The wheels which he left unfinished were completed by a melancholic man, who has been in confinement twenty years, and who, since building the water-cart, has been daily employed in the carpenter's shop, and has undergone very great improvement, both mentally and bodily.

"The first patient admitted into the asylum is especially remarked upon in my former report: he is now an apt scholar in learning shoemaking: and another patient, formerly a schoolmaster and toll-collector, receives instruction in the same trade. Two of the males who have been discharged cured, after being employed in the garden, were encouraged to resume their business, and recovered whilst working under the direction of the shoemaker attendant. Two other shoemakers are convalescent, one of whom was mute and obstinately refused his food for eleven months, during which period strenuous efforts were repeatedly persevered in to induce him to work, which have proved eminently successful. From having been extremely attenuated, he is now in good health, is intelligent, and voluntarily enters into conversation. About two months ago, he accompanied the shoemaker attendant to York, where he selected with judgment a supply of leather for future use.

"A female patient, who became insane after a faithful servitude of seventeen years in one family, and who was very riotous for the first three months, and laboured under peculiar religious delusions, was allowed to fulfil the duties of under laundry-maid. The high character she bore for integrity was an inducement for permitting her to share the privileges of the servants: this partial restoration to a condition similar to the one she had previously filled for so many years, was the means of tranquillizing her. During this probationary treatment, she visited York in company with a servant, and was at liberty to walk about the grounds at stated times; the ordinary confinement to wards and airing courts would, in my opinion, have established her insanity. Five months ago she was discharged cured, and has since acted as housekeeper to a widower brother's family."

"A lad brought from another Asylum, shortly after the opening of this, in a state of dementia, is learning to be a tailor. His growing intelligence induces the hope that he will be enabled some day to earn his own living. His companions are equally interesting, one of whom was formerly a tailor, has been in confinement six or seven years, and is reduced to a state of idiocy: he is improving slowly, and occasionally reads aloud to other patients. Seven patients have been useful in the blacksmith's shop, although only two of them belonged to that business. Two who partially acquired the trade have left the Asylum, recovered. In every instance of patients working at trades, cures have been effected; amongst the first were a tailor and bricklayer. The basket-makers have been under charge of the gardener attendants, and have plied their business in the garden-house. Whenever patients are employed as stone-masons, they are placed under the charge of the bricklayer."

The following remarks on the treatment of suicide cases will be read with pleasure :—

"It has not been our practice to deprive patients of that class of the use of implements required in their occupations, with which they might have effected their desire under less vigilant and careful watching, because it is believed that whenever the tendency is lasting, the chances of overcoming and wearing out the disposition to commit suicide, are more remote when patients so afflicted are studiously prevented handling instruments, than when, under strict watching, they are permitted the use of them : the greater the familiarity with their particular purposes, so much less is the wish to use them improperly. Some marked and successful cases have justified this plan of treatment. Such a practice at first sight may seem to be attended with risk; I think it is not so much so as is imagined. A patient inclined to destroy or mutilate himself is placed under the care of an attendant, accompanied by a few other patients not similarly disposed; they are all employed, and have tools amongst them with which mischief may be done. The attendant, aware of the individual propensities of his group, arranges them accordingly, and so places the dangerous patient that he may be constantly under his own observation and that of the more intelligent of his workmen.: it can scarcely happen that an opportunity will occur for the suicidal patient to accomplish his aim. As time goes on, the continuous useful application of the implement, whether hammer, knife, scissors, chisel, hatchet, scythe, or any other tool convertible into a weapon, reconciles the patient, inspires him with confidence and hope; his enervated mind acquires strength, his broken spirits are recruited, and he cherishes the feeling that he will be again restored to society. The waverings and instabilities of disordered minds, and the sudden impulses to which they are liable, are alone to be comprehended by experience in their management. When understood, I do not believe that the danger to this very pitiable class is so great under active employment, as when they are merely detained or engaged within the wards and airing courts : the chief number of suicides in lunatic asylums have been committed within the walls, where it was supposed very little, if any, opportunity could be given for the perpetration of such acts."

On the subject of medical treatment it is observed,—

"Out of the fifty-five new cases admitted, there have been several of each sex received in a state of raving madness, attended with excessive violence, in all of which, during the continuance of the maniacal paroxysm, I administered port wine and porter, besides a liberal diet in the form of good broth, eggs, and other convenient kinds of nourishment; tonic medicines were employed for some, but the resistance offered frequently does more harm than the remedies do good, which does not so much happen when nutriment of an enticing description is presented to the patient. No case has occurred in which any depletory measures have been adopted, either by bleeding or the use of antiphlogistic medicines, save aperients. Inflammatory action in the brain seems to be a disease of very rare occurrence in acute mania; the physical powers are monopolized for the full development of the disorder in its genuine form; its character would be incomplete if the energies of the constitution were diverted for the establishment of inflammatory action, and raving madness would probably be a rare phenomenon : it is an infliction so singular in itself, requiring so much strength to portray its true existence, and so often making attacks in asthenic conditions of health, that the whole business of the maniac's enfeebled frame is to demonstrate the malady. Nature is slow either to set up or sustain inflammation of the brain, or of its membranes, whilst the physical powers are so intently engaged in maintaining the peculiar violence of madness. The indication, therefore, is to succour the constitution, and thereby to fortify it against the debilitating influence of long continued raving ; an opposite plan of treatment renders the heart irritable by deranging the digestive organs, prolongs the paroxysms, delays repose, checks and postpones recovery, if the results are not fatal, nor end in confirmed dementia. Examinations after death show a want of blood in the brain, a deficiency of firmness in its substance, frequently too much water in the ventricles, and some at the base. I am speaking particularly of the post-mortem appearances after acute mania, and do not refer to its complication with other diseases to which the insane are liable. I shall, perhaps, be better understood if I allude to the case of a man suddenly seized with madness accompanied by violence, or of a lying-in woman attacked with mania ; such patients are seldom the subjects of inflammatory action of the brain, any more than the individual who bursts into a violent fit of passion, and whose reason and judgment are in abeyance only, whilst the heat of temper lasts."

It appears, from the eighth report of the medical officers of the *County Lunatic Asylum, Forston, Dorset,* that since the last Report the number admitted has been eighteen males and thirty females.

"Of this number, four males and ten females were received within three months after the first accession of the disease. This is a larger number of recent cases than was received during the previous year. It should, however, be observed, that several of these cases were suffering from paralysis, or epilepsy, or other diseases tending to shorten life. Two were admitted after three, and within twelve months from the commencement of the disorder; twenty had been afflicted for periods varying from one to twenty years; and in seven of these, the malady was associated with epilepsy or general paralysis. Twelve were cases of recurrent insanity.

"In thirty-one of the whole number of cases admitted during the year, the mental disorder was accompanied by physical disease; such as cerebral and hepatic congestion—paralysis—epilepsy—apoplexy—impairment of the digestive organs—disease of the heart—or derangement of the general health—leaving seventeen cases where the mental malady was not complicated with obvious and extensive physical disease.

"Hereditary predisposition existed in ten of the cases admitted."

The following remarks on the importance of *early treatment* in cases of insanity, cannot be made too generally known:—

"The past year evinces that there is still much procrastination in conveying the insane poor to an asylum, in consequence of which, it is much to be feared, the disease is aggravated, and often proves fatal. Thus wives and children are too frequently deprived of their natural protectors, and compelled to apply to their parishes for relief, and, in not a few instances, become a severe burden on the rates. One inference to be drawn from the statistics of insanity is, that a large portion of those individuals who are withheld from an asylum during the early period of the disease, become the subjects of chronic insanity. The expense incurred in maintaining the destitute insane at the commencement of the malady, would ultimately prove a measure of pure economy. In many instances the expenses of a few months only would fall on the parishes, instead of their becoming, as is too often the case, paupers for life, and with the miseries of the unfortunate patients greatly aggravated. There is much reason to fear that, unless measures are adopted more efficient than those at present resorted to, great neglect will continue to exist. A well-devised system of immediate transmission, with early treatment, might probably have arrested the ravages of the disease, at least among those who had not, from hereditary tendency or physical disease, a strong predisposition to the malady. It would, perhaps, in a measure mitigate the evil complained of, if all the boards of guardians would provide the parochial officers with the Act relating to Pauper Lunatics, of the 8 and 9 Vict.

"While the patient maintains an apparent consistency of conduct, and coherence of thought and speech, the friends seldom have any suspicion of the existence of mental derangement. The changes in the disposition and temper, to which, in reality, the greatest importance ought to be attached, are generally overlooked, or they attract very little attention, and are regarded only as something not quite right. During the stage of incubation, the mind is frequently abstracted from that which is passing around, brooding over some idea which has taken possession of it. The expression of the countenance is changed, the eye passing from object to object, without fixing on any. The attention is with difficulty arrested, and when the sound of the voice has ceased to vibrate on the ear, the patient relapses into his former stupor; he moves as if mechanically, his dress is perhaps neglected, and he seems to have no desire to please. If questioned on the subject, he will generally acknowledge that he suffers severe pain, or a sense of weight in his head, and the frontal region, or the top part of the head, is generally referred to, as the seat of the uneasiness. His nights are often disturbed, and very little sleep is obtained. These symptoms continue for a longer or shorter period, but as they differ from the ideas generally entertained of the incipient signs of mental disease, they give rise to no suspicion of the real cause until some untoward event takes place, an outbreak perhaps of maniacal excitement, when the utmost astonishment and alarm are created. The flushed face, the bright and suffused eye, the violent gesture, incoherence of ideas, and maniacal expression of countenance, which are the symptoms looked for by those who entertain the popularly-received notions of insanity, may be, and frequently are, altogether absent."

Again, when speaking on the subject of premature removal from medical treatment, it is observed,—

"When the admission of the patient into the asylum has been obtained, the friends immediately become impatient for the results; and where they have, as is sometimes the case, to defray a part of the expense of the maintenance, they become weary of the charge. They cannot believe that the persons to whose care the insane poor are committed, have no interest whatever in keeping them in the asylum longer than is for their own good. If, on visiting them, they find that they can converse rationally on ordinary topics, and are well-conducted, they consider them quite well: and if, in addition to this, they are informed the patients assist in the wards or the laundry, or that they are daily occupied in the field, the cure is no longer doubted, and it is positively affirmed that they ought to be discharged. The parish officers are, without delay, put in possession of the supposed fact, and letters are despatched to the superintendent or to the visitors, urging the immediate discharge of the patient. The above-named parties are incapable of forming a correct judgment on the real condition of the brain and nervous system, and whether those organs have become sufficiently restored to allow the patients to engage without detriment in their former pursuits, and to take an active part in society. If patients, at the commencement of convalescence, are prematurely removed, a speedy relapse may be the consequence; while if permitted to remain for only, perhaps, a short time longer, permanent recovery may be secured. In consequence of patients being removed before their recovery is fully established, the benefits and utility of institutions for the treatment of the insane are decried: and the imagination of the patients being still disordered, they sometimes circulate erroneous reports; the worst effect of which is to deter other persons, who might probably be restored, from being sent to an asylum. As soon as the patients are considered to be so far recovered as to justify their discharge, it takes place as a matter of course. It is a source of no small anxiety to the mind of the physician, to determine when convalescence has really taken place; and his perplexity is considerably increased by constant importunity, both by letters and visits, of the friends and the parish authorities. It is important that on this subject the public should entertain correct views. In many cases, however, it is much to be feared that a false notion of economy, in a saving of present expense, is often of great weight in the consideration of the persons on whom the relief of the poor devolves."

The Report speaks of a female patient, who had such a morbid craving for spirits, that—

"Even the odour of it would create an intense desire to drink, in which she indulged until completely overcome by its effects. Yet she is fully aware of the consequences of her sad propensity, and bitterly deplores her infatuation; she will frequently exclaim, with apparent grief, 'Ah, drink has brought me to this!' She has a sister in the asylum, who was admitted from the same cause. Both were paralytic, one in the right arm, and the other in the left. It has been subsequently ascertained, that the father of these females led a very intemperate life, and that he died a confirmed drunkard."

On the important subject of the occupation of the insane the medical officer judiciously observes,—

"To draw the insane out of themselves, to engage their attention, and to induce them to follow any useful occupation, even in the most trifling degree, is in many cases attended with difficulties, of which persons unacquainted with the subject can form little idea. The advantage is often so obvious, even to the patient himself, that when once an interest has been excited, he will voluntarily engage in the accustomed labour, as a means of relieving his mind from the troubles which oppress him. This, however, is far from being always the case. It has, in several instances during the past year, as in former years, been necessary for an attendant to remain by the side of the patient, and to direct his every movement for days, and even weeks ; and the results have fully recompensed the time, attention, and perseverance bestowed. Some thus aroused from their state of abstractedness, have become cheerful, and have ultimately been restored to their families ; of which the following is a striking case. A man who had formerly been a master shoemaker, from severe losses, had become reduced in his circumstances. He had sunk into such a deplorable state of apathy, that he had not power to measure for a pair of shoes. Having one day, in a paroxysm of despair, attempted to terminate his existence by cutting his throat, he was sent to the asylum. When admitted he suffered from disorder of the liver. After a short time he was prevailed upon to commence work, and when he had finished a pair of shoes, he observed it was the first pair he had made for twenty years, having been accustomed to cut out only. He has since been discharged."

Again, it is wisely said,—

"In common maniacal cases, instead of the nervous excitement expending itself in the indulgence of mischievous propensities, in quarrelling and vociferation, it is carried off in useful labour. In the case of the melancholic, the relief afforded renders him quite a different being ; and instead of brooding over his imaginary crimes or real miseries, he is comparatively cheerful and happy. Let the system of employment in asylums be discontinued, and they would speedily become mere prisons for the custody of the insane—scenes of dulness and listlessness on the one hand, and of numerous accidents and constant confusion on the other; entailing an increase in the expenses, and an additional number of attendants, or a return to the worst forms of coercion and bodily restraint."

The Report of the Medical Officers of the *Dundee Royal Asylum* is ably drawn up, and presents to the psychologist much suggestive information. How gratifying to hear the non-restraint system of treatment thus spoken of :—

"Here for many years past restraint has never once been thought of, nor is the want of it ever felt to be of the slightest inconvenience. Blessed be the memory of that enlightened Frenchman who thus banished from medical science its greatest reproach, and instead of chains, manacles, and stripes, gave to the unhappy maniac the most soothing indulgences and a comparative Elysium !"

The following remarks on the epistolary correspondence of the insane with their relatives is deserving of attention,—

"Several well educated patients carry on a constant epistolary correspondence with their relatives at home and abroad. This is at once the means of occupation and amusement, and they take much pleasure in it. Nothing can exceed their delight, when they receive letters written in a kindly strain in return. The soft and soothing answer operates delightfully. In their case it seems to be always '*balm to the hurt mind.*' The intercourse, when thus gently and judiciously conducted, generally contributes either to the relief of the patient's malady for a time, or to its ultimate cure. On the other hand, there are patients whom letter-writing would injure, and in their cases, we need scarce add, it is not allowed. Among the epistolary gentlemen, there is one who distinguished himself this year by drawing up the report of our last festal anniversary for the newspaper. We have still amongst us, too, the three gentlemen who were referred to in last year's report, as having contributed so much both to their own happiness and the amusement of the establishment, by their diverting and witty facetiæ."

Towards the conclusion of the report, Dr. Mackintosh, the late superintendent, bids a final adieu to his colleagues, having received the appointment of chief medical superintendent in the Royal Glasgow Asylum. He speaks in terms of high commendation of all those associated with the Dundee Asylum. He was much liked by the patients and the officials. It will be difficult to replace him.

The eleventh report of the *Suffolk Lunatic Asylum*, for 1849, is now before us. The following will give our readers an idea of the state of the house in 1848:—

| | Males. | Females. | Total. | Males. | Females. | Total. |
|---|---|---|---|---|---|---|
| In the House, December 31, 1847 | | | | 116 | 134 | 253 |
| Admitted since | | | | 31 | 51 | 82 |
| | | | | 147 | 185 | 332 |
| Discharged, Cured | 13 | 25 | 38 | | | |
| " Relieved | 0 | 2 | 2 | | | |
| Died | 11 | 16 | 27 | | | |
| | | | | 24 | 43 | 67 |
| Remaining in the House, Dec. 22, 1848 | | | | 123 | 142 | 265 |

The particulars of the following case will prove interesting to our readers:—

"E. B., a female, discharged on the 12th of August last, was re-admitted on the 18th of October. Throughout the whole time of her absence (about nine weeks) she had been as reported, perfectly well, till a few days previous to her return. There is hereditary pre-disposition, her father having died in this Asylum in April last. Since her discharge in August, her quiet and contented manner was noticed by several of the domestics of the house, with whom she continued more or less intimate; she was full of expressions of gratitude for her own restoration, and interest about other patients she had left behind. She had been very actively employed, and gave general satisfaction to her employers. The immediate cause of her return was a self-accusation of having poisoned her brother, and declaring that she had done so with the desire to be hanged. This led to a judicial inquiry on the death of this boy—a child fourteen years of age, and somewhat feeble in mind and body. There was no evidence on which she could be convicted, though her own confession was persisted in; and now she talks of the act with an hysterical laugh, but says she only wanted 'to get accused of the intention.' Now, supposing her self-accusations to be true, the disposition is rather *suicidal* than *fratricidal.* Morbid impressions may exist for months, and perhaps years; during which time, on renewed investigations, self-accusing tales may be so plausibly told as to mislead many: and in this case there was none of the sullenness and apparent stupidity which may characterize an irresistible impulse urging on to bloodthirsty destructiveness. Just before her former admission, she had tried to destroy herself and failed; and a few days before her return, she had attempted to throw herself into a river, but was prevented: and these repeated failures urged her to charge herself with the destruction of her brother, as suggesting the next easiest mode of *self*-destruction. This is the suicidal impulse, and the impelling motive of this imaginary act is the desire to experience its known consequences. After other failures, this suggests itself as the readiest means for the accomplishment of the end she desires."

It appears from the Report of the *Bedford Lunatic Asylum,* 1849, that "the number of patients remaining in the Asylum at the close of the year 1847 was 206—107 males, and 99 females." One hundred patients are said to attend divine service. The combined reports of visitors, medical officer and chaplain, are extremely gratifying. The asylum is represented to be in excellent working condition.

The nineteenth report of the *Belfast District Asylum* (1849), contains considerable valuable statistical data connected with the condition of the insane patients in the asylum. In speaking of one of the criminal lunatics, the report says—

"He had been in the house nearly nine years, during which time he conducted himself so quietly and properly as to make his great mildness, propriety, and docility, in general, strangely to contrast with the dreadful tragedy he had enacted, four lives having been sacrificed by him,—viz., those of his wife, two children, and mother-in-law. Throughout his inmateship, he never expressed the least wish to be at liberty, and no remorse in particular had ever been manifested by him for what he had done: when any allusion, however, happened to be made to it, he appeared ill at ease; but still, the utmost that ever could be collected from him, respecting an occurrence so truly shocking, was, that being unconscious of the whole proceeding, at its commission, he hoped he would not be held accountable for it in a hereafter state."

In speaking of the suicidal and homicidal cases, it is observed—

"Twenty-three of the patients admitted this year were suicidally disposed—viz., ten males and thirteen females—all having made very determined attempts at self-destruction, prior to admission. Those attempts were as follow:—By drowning, eleven (six males, five females); cutting the throat, eight (two males, six females); hanging and strangulation, two (one male, one female); cutting into the abdomen, one (male); poison, one (female). In sixteen of the admissions, suicide had been threatened or meditated, but not actually attempted—viz., fourteen males and two females. The homicidally disposed cases amounted to four (males.)

"It is a matter of great thankfulness to have to state, that amongst such a number of inmates as the above, all having so great a propensity either to injure themselves or others, no accident, or other untoward event occurred in the establishment, during the year."

We now propose bringing under the notice of our readers, the valuable report of Dr. Browne, of the *Crichton Institution, Dumfries.* Of the one hundred and thirty-seven applications for admission, it appears that "twenty-two males and nineteen females have been received, making a total of 196 patients resident. Of this number sixteen have died, twenty-five have been discharged, twenty as recovered and five while under treatment."

When speaking of the influence of the great social changes which have taken place in society on the Continent, Dr. Browne observes—

"It is probable, that much of that unsoundness of mind which cannot justly be attributed to the grand revolutions for good or evil in which mankind is involved, nor to any of those events which disturb the surface of society, may be found to originate in that profound and universal activity which characterises the intelligence of the present generation. It is very obvious that the multiplied means, and the rapidity with which new thoughts are transmitted and adapted to the comprehension of the ignorant as well as the educated; that the incessant reception of new and powerful impressions; the deep and intense interest which now attaches to every question; the daily appeals to passions and sympathies; the craving for novelties, discoveries, and for rapidity of moral progress, must all tend directly and inevitably to establish a condition allied to morbid excitement, exhausting to the system, open to the incursion of disease, and incapable of resisting the effects of disappointment and misfortune. It is not a witticism, but a grave and pregnant truth, to affirm that an individual actuated by the cares and pleasures and pursuits of the current century, 'lives more of life,' passes through more numerous, more vivid, and more perilous phases of intellectual existence in one year, than his immediate ancestor did throughout his whole career. While it does not always happen that the symptoms of mental alienation bear any relation or resemblance to its cause, that fear should produce fear, yet it has been observed that the feverish activity, the unreasonable if not Utopian ambition, and the constant tension of the powers described, is very generally followed by depression, despondency, and despair. In the group under consideration, there are youths who have passed years amid all the rapid alternations and eager hopes of railway speculation, in the construction of aërial machines, in the propagandism of a new political faith; there are women in the prime of life who now, in all probability, experience the results of that fervour of devotion, but fickleness of principle, which seeks gratification in powerful, even although painful, impressions. The desire to acquire fortune or fame rapidly, even suddenly, is but a modification of the same spirit which inspires the desire to communicate knowledge rapidly by a royal road, by a tax and strain upon the energies, or by saving them all exertion and application. When directed towards the young, it fosters that precocity which is naturally engendered by the tendencies of society, by the modes of life, by the very recreations of the play-ground, and favours the development of disease. It stimulates in the cradle; it anticipates nature; it imposes the burden of maturity upon the feebleness of infancy, and kindles those tendencies which so often terminate in scrofulous or nervous disease. Statisticians believe that the age at which insanity appears is now earlier than it was fifty years ago; and although other causes must contribute to such a result, there is abundant evidence to prove that prematurity of intellect, whether natural or superinduced, tends to early decay. Several illustrations of these observations have occurred during the present year. One, himself a teacher, trained under a system which enforced continuous application, enjoined an hour's relaxation and eleven of study, or mental attention of some kind, and openly professed as an object to condense the greatest possible amount of knowledge in the shortest possible time, is now exhausted, dejected, dispirited, aged, and decrepit in mind, but deluded into the belief that he has accomplished all things. A gentle girl incessantly craves a Testament in the original language, which she acquired in conjunction with an amount of Biblical and varied learning more calculated to exercise the memory than to strengthen the understanding. In a third, the muscular system seems to have been forgotten, and the fine proportions of an athlete are associated with the accomplishments of a dilettante. It would be rash to affirm that education evoked this ruin. The capacities, the temperament, and the hereditary tendencies of the patients may have been such as to endanger the stability of reason; but to the injudicious application, the amount, or the selection of the means of training, may be referred the nature and the period of the catastrophe."

Dr. Browne defines melancholia to be "misery with delusion." Are delusions always present in true melancholia? We doubt it. Cases of intense melancholia have come under our notice and care, in which we could not trace the faintest semblance of delusion of any kind. Dr. Browne says, most probably of these cases,—

"There may be no apparent delusion, no assignable motive or object; but the patients fear all things—their own thoughts, solitude, society; they fear their own fears; or their face is pale, their limbs tremble, or they weep and shrink, or they sit downcast and motionless from the mere exaltation of painful emotions which they cannot subdue, and which neither reason nor sympathy can reach or mitigate. They envy those who suffer less, even although this immunity be purchased by imbecility and stupor. They feel—

'Wretched capacity of frenzy, thought!
Wretched capacity of dying, life!'

They are often able to reason upon these groundless terrors; to admit that they can neither explain nor justify their sufferings; that the arguments used to prove their fallacy are conclusive and unanswerable; but they continue to despair. There is a disease of the will as well as of the emotive powers. An old man who labours under this affection, flies to the asylum in order to escape from his despondency, and has recently left his place of safety with the determination to return should his tranquillity be again disturbed. So exquisite and intense are these feelings, as to urge the sufferer into acts of suicide and violence. One of three patients admitted with this form of alienation, but whose mind was previously impaired in strength, attempted to murder a sister while labouring under the frenzy of fear; and there is every probability that many of the sudden precipitations from cliffs, windows, and bridges, are to be explained in a similar manner. In many of these cases there are present disease of the heart and large vessels, which from their effects upon the phrenic plexus may produce, as they are known to be accompanied by, internal sensations of distress and disturbance, which have received the name of anxiety, and certainly affect the mind with apprehension. The fear of death exists in many men in sound health, independently of delusion; a feeling of depression is often experienced and indulged in youth which may be connected with the cause, and are certainly premonitions of nervous disease."

On the subject of the sleeplessness of the insane, it is observed—

"When this morbid acuteness is combined with timidity, suspicion, or spectral illusions, the will is exerted in repressing the inclination for rest and unconsciousness. The patient dare not sleep, as death, or his persecutors, or a phantom, may surprise him during slumber. There is, however, a state of the brain which appears to be incompatible with sleep. Nocturnal restlessness is a symptom of all varieties of insanity in its acute stages; and all lunatics, with the exception of the imbecile and of confirmed monomaniacs, who engage in labour, occupy many of the hours allotted to sleep in noise or reverie; but protracted vigilance is chiefly observed in lypemaniacs. There is a patient whose mind was ruined with his fortunes, who has resided in the House for about a year, and has rarely been seen to sleep, although from his enfeebled and critical condition, the visits of the night guardian are numerous, and made for the purpose of observation. That this restless anxious being does sleep is certain; but it is manifest that his slumber cannot be profound, that his senses and watchfulness are still partially awake, and that notwithstanding the precautions of those around, every sound reaches his ear and rouses his attention. Another individual of the same class, who is urged to desperation by terror, who converts every object into a source of alarm, for many months spent one or two hours of the night in sleep, and the remainder in cries of terror, or attempts to escape from the dangers with which his apprehension peopled his room. Latterly sleep seemed to forsake him altogether; the pervigilium resisted narcotics so obstinately, and so detrimental to his health were his wanderings through his room, his struggles with apparitions, or with his own conscience—for he does not reveal the grounds of his alarm—and his efforts to obtain security, that constant watching has become necessary. It has been resorted to upon the principle, that companionship may soothe, may inspire the feeling of protection, and induce him to remain quiet in bed, and that a cheerful fire and light may dispel the imaginary horrors which darkness conjures up."

In speaking of the insensibility of the insane, Dr. Browne says—

"This torpidity of the nervous system is chiefly manifested in melancholic females. Suicide, and self-mutilation of the most cruel and appalling kinds, have been practised; the religious fanatics, called the Convulsionaires of St. Medard, bore with pleasure and relief to the hysteric ecstasy into which they were thrown, the infliction of every species of torture. Cases occur in every Asylum of complete anæsthesia, in which operations have been performed, and pain applied therapeutically, by blisters, cupping, &c., and no cry or confession of uneasiness been elicited; where diseases attended by suffering, even by excruciating agony, have advanced to a fatal issue unnoticed, perhaps unknown to the victim, showing that even the ganglionic feeling, which is exalted in many other examples of melancholia, is here suspended or impaired. There is among the melancholics an experimental suicide, who has tried upon his own person various means to extinguish life, partly to determine the comparative merits of the different physical modes of escaping from moral disquietude, and who was only prevented from accomplishing his purpose, by strangulation with his own hands, in consequence of the loss of consciousness."

We have been favoured with the Twenty-Second Annual Report of the Directors of *James Murray's Royal Asylum for Lunatics* (1849.) This asylum was first opened on the 28th of May 1847.

It appears that since the asylum was first opened, to the present time, 748 patients have been admitted—389 males, and 359 females. Of that number 110 were re-admissions; and if these are deducted from the gross number just mentioned, the total number of admissions would be 638—347 males, and 291 females. Out of this number, 313 have been cured—138 males, and 175 females; 111 have died—70 males, and 41 females. Seventy-one have been removed much improved; and many of whom, had they been allowed to remain long enough in the house, would, in all probability, have been also cured. Eighty-six have been removed with little or no improvement, the disease appearing to become permanent, and the friends or relations of those persons either took them home to their own houses or sent them to other places.

The number of cures as compared with the admissions presents a most cheering and delightful fact to all well-wishers of their species; and while it pronounces a marked encomium on the efficiency and success of this Institution, it proves beyond dispute that the curative processes which medical skill has devised and applied in the treatment of the insane are productive of the happiest results.

It further appears from the Physician's Report that intemperance must be classed among the principal exciting causes of insanity, a fact which cannot be too generally known. This is not to be understood as applying merely to cases of *delirium tremens*—the immediate effect of the abuse of intoxicating liquors—but is also the consequence of a long course and continuation of dissipated habits, which, by gradually creating bodily disease and infirmity, lays the foundation of dyspepsia, sleeplessness, and other symptoms premonitory of insanity.

During the past year, 37 patients have been admitted—21 males, and 16 females; 18 have been dismissed cured—8 males, and 10 females; 9 have been removed by their friends, more or less improved —7 males, and 2 females; 4 have been removed unimproved—2 males, and 2 females; 8 males have died, and there now remain in the house 162 patients—90 males, and 72 females.

The system pursued in the treatment of the insane in this asylum has invariably been gentle and practical—studying throughout to make their position, as nearly as circumstances would permit, as comfortable as possible, that they might be less sensible of the deprivations to which they are unavoidably subjected by being removed from home. All the courtesies of life are observed in intercourse with them, they have free ingress and egress to such of the airing grounds as their malady and situation in life render proper and fit for them, and these privileges they enjoy very much. They are free from personal restraint, in so far as this is beneficial and safe for themselves and others, and live altogether in their different wards and galleries like a large family.

Among the prevailing causes of insanity, Dr. Malcom ranks hereditary predisposition. He says :—

"In the great majority of cases, I have been able to elicit this truth; but I can, at the same time, say that I by no means find the disease is less easily cured when this is the case, nor do I consider it a reason for a more unfavourable prognosis. I may also incidentally mention, that insanity, like gout and consumption, or any other hereditary complaint, may, and does wear out in succeeding generations, and leaves the offspring of persons who have suffered from these diseases free from them altogether. Intemperance holds the second place as the exciting cause of insanity. By this I do not mean to include cases of delirium tremens—the immediate effect of the abuse of intoxicating liquors—but insanity arising from a long course and continuation of dissipated habits, which, gradually creating bodily disease and infirmities, lays the foundation of dyspepsia, sleeplessness, and those symptoms premonitory of insanity. Poverty has in all ages been a fruitful cause of insanity; the long continuance of privations, the frequent want of the most common necessaries of life, famine, and the dread of approaching famine, have driven many to the commission of suicide or murder, or both. These are the immediate effect of that sudden and irresistible insanity which seizes the mind, and the result is as frequently sudden and violent. But there is another, and what I would call a lesser, insanity which attaches itself to those unfortunate individuals: a species of slow delirium gets possession of the mental faculties, during which the person attacked gives utterance to the most preposterous chimeras and imaginations, saying that they are possessed of devils, have intercourse and receive support from devils, to whom they have sold themselves for a period of years, and to whom they have made over their eternal salvation; that, by the powers thus granted to them, they have the means of injuring, or as their phrase goes, of bewitching those to whom they have spite, or an ill-will: at other times they declare that they have had carnal connexion with witches, wizards, or incubi. Strange that these hallucinations should have been believed by wise men, judges, clergy, physicians, and the learned of their day; and how these men could admit as facts, things so perfectly in opposition to the laws of nature, condemning thousands of the unhappy maniacs to be burned as witches, when a more humane and more enlightened policy would have sent them to a madhouse. It is worthy of remark, that this species of insanity prevailed in all countries nearly about the same time—all over the Continent, as well as in England and Scotland; and the crimes and legal murders perpetrated by our forefathers amounted to a number at which we now shudder to think. It would extend this report too far were I to pursue this most interesting inquiry. I will therefore merely state that the great, very great majority of those persons who suffered for supposed witchcraft, were either poor old women worn out with sufferings and hunger, or young women suffering from hysteria and uterine diseases."

In the Report of the *Montrose Lunatic Asylum* (1849), we find the following tabular statement, showing the general results of the year:—

| | Males. | Females. | Total. |
|---|---|---|---|
| Remained in Asylum .................... | 70 | 62 | 132 |
| Admitted during the year .............. | 17 | 23 | 40 |
| Total under treatment ......... | 87 | 85 | 172 |
| Discharged, Recovered.................. | 7 | 7 | 14 |
| „ Convalescent ............ | 1 | 1 | 2 |
| „ Unimproved ............... | 2 | 0 | 2 |
| Removed ................................ | 1 | 0 | 1 |
| Died ....................................... | 7 | 4 | 11 |
| | 18 | 12 | 30 |
| Remaining 1st June, 1849,........... | 69 | 73 | 142 |

The account which Dr. D. M'Gavin gives of the present condition and future prospects of this asylum is extremely satisfactory. Reference is made in the report to a patient in the asylum who, in consequence of refusing food, was obliged to be fed by means of the œsophagus tube. A somewhat singular circumstance connected with this case was related some weeks after the patient's admission, by the ordinary medical attendant, who is a highly respectable practitioner in the town, and which furnishes another and rather a singular use to which that wonderful agent (chloroform) may be applied. The patient, while under this gentleman's care, had obstinately refused food for some time. After every inducement failed in accomplishing the desired object, the use of the stomach-pump was determined on, and at this moment it happily occurred to the Doctor that chloroform might be serviceable. Food and medicine being prepared, the patient was plied with the chloroform; and while partially under its influence he ate and drank heartily everything put before him, laughing and cracking jokes the whole time. The change altogether produced in the mind of the patient was most gratifying. The Doctor, and a clergyman who happened to be present at the time, and whom he had previously viewed with the utmost suspicion and dislike, he shook warmly by the hands, and acknowledged, in unmistakeable terms, to be his best friends and protectors. In all cases of refusal of food, if its application be equally successful as in that just now described, a decided boon will be conferred on that class of patients, on many of whom a painful, and sometimes dangerous operation requires to be performed, with a view to the preservation of their lives.

The Report of the *West Riding Pauper Lunatic Asylum*, (1849,) contains a detailed account of the *cholera* outbreak, which carried off so many of the patients; but it is consolatory to find that, with scarcely an exception, the patients carried off have been subjects of incurable mental derangement; and in a majority of instances so demented as to be unable to describe their symptoms, while many perversely refused alike food and medicine. A large number of the fatal cases appeared suddenly death-struck, without premonitory symptoms of any observable kind, and sank in from eight to twelve hours: in others the attack was more prolonged, and some survived two or three days. The most rapid case was that of an idiotic girl, (*Hannah Sutcliffe*), who had gone to bed in apparently good health on the evening of Friday, the 26th: she was sleeping tranquilly at three A.M.; was attacked with cramp and collapse at half-past three; became immediately cold and livid, and expired at half-past seven, after an illness of only four hours.

With respect to the causes of this terrific outbreak in the Asylum, the visiting physicians are as yet unable to offer a decided opinion. The first cases seemed attributable to infection brought by the patient from Gomersal; but subsequent attacks occurring, almost simultaneously, in all parts of the Institution, remote from and unconnected with previously infected wards, are more difficult of explanation. The medical officers, however, are pursuing their inquiries, by collecting and analysing all the professional details relative to the disease, which the urgency of their several engagements has left them leisure to observe and record, with an endeavour to arrive at a more definite solution of this, and other debated questions, in the history of this awfully interesting disease.

It appears that between Oct. 2 and 31, eighty-five patients died of cholera—49 males, and 36 females.

